# Complete sequence of the closed circular extrachromosomal element (CERE) of *Naegleria australiensis* De Jonckheere (strain PP 397)

**DOI:** 10.1128/MRA.00321-23

**Published:** 2023-09-26

**Authors:** Brian T. Nguyen, Nora M. Chapman, Niklas Johnson, Holly A. F. Stessman, Steven Tracy, Kristen M. Drescher

**Affiliations:** 1 Department of Medical Microbiology and Immunology, Creighton University, Omaha, Nebraska, USA; 2 Department of Pathology and Microbiology, University of Nebraska Medical Center, Omaha, Nebraska, USA; 3 Department of Pharmacology & Neuroscience, Creighton University, Omaha, Nebraska, USA; University of California, Riverside, California, USA

**Keywords:** *Naegleria*, CERE, rDNA

## Abstract

Ribosomal RNA is not encoded in chromosomal DNA in amoebae of the Naegleria genus but the rRNA genes are located on closed circular extrachromosomal ribosomal DNA (rDNA)-containing elements (CERE). In this report, we describe the sequence of the CERE of *Naegleria australiensis* De Jonckheere (strain PP397).

## ANNOUNCEMENT

Amoebas of the *Naegleria* genus have no ribosomal DNA (rDNA) in the nuclear DNA but rather in the nucleolus (1–4) on closed circular extrachromosomal elements [CERE (5) ]. One trophozoite contains ~4,000 CERE (1, 2). CERE also contain a large non-ribosomal sequence (NRS) with a single origin of replication (6). Unlike highly conserved rDNA cistrons, the NRS show limited conservation between *Naegleria* species (5). *Naegleria australiensis* De Jonckheere (PP397) was isolated from drainage water in Australia in 1973 (7) and is not pathogenic for humans but in mice induces brain pathology like *Naeglaria fowleri* (8) as well as pneumonitis (9).

*N. australiensis* was obtained from ATCC (Manassas, VA) and trophozoites cultured in modified PYNFH (peptone/yeast extract/nucleic acid/folic acid/heme with 10% fetal calf serum) medium at 25°C and split every 48 h. CERE DNA was isolated using the Plasmid Mini Kit (Qiagen), electrophoresed on 0.8% agarose gels, and unnicked supercoiled DNA purified using the Monarch Gel Extraction Kit (NEB). As known, *Naegleria* CERE sequences have numerous repeat regions (5); CERE were digested with *PstI* (NEB) in lieu of shearing the DNA, resulting in a product of approximately 12–13 kbp. All remaining processing/sequencing was performed at the Roy J. Carver Biotechnology Center (University of Illinois—Urbana-Champaign). The Agilent Femto Pulse System was used to determine the DNA quality. The PacBio SMRTbell Express Template Prep Kit 3.0 (Pacific Biosciences) was used to prepare the library, and the PacBio Sequel IIe (SMRT Link v11.0) was used for sequencing in CCS mode and 30 h movies.

The 130,147 raw reads were filtered against the mitochondrial sequence of *Naegleria gruberi* (GenBank accession NC_002573.1), and sequences with at least 70% identity (33,735/25.92%) were removed from analysis (E-value 1e-50). Due to the close identity of rDNA between *Naegleria* species, a BLASTn alignment was executed on the outstanding 96,412 reads against the *N*. *gruberi* rDNA (GenBank accession AB298288; BLASTn parameters: >75% identity to the rDNA sequence, E-value 1e-50). A total of 51,009/96,412 (52.90%) sequences with rDNA identity were retained for analysis. These 51,009 reads were separated into FASTA files with read between 9 and 15 kbp (5,894/51,009; 11.5% of reads), based on the predicted size of the CERE. Reads were assembled using the SPADES Assembler 3.15.5 into contigs and a consensus (options: “—isolate,” “—only-assembler”; K-mer-125). Characteristics of the CERE are presented in Table 1.

**TABLE 1:** CERE characteristics of *Naegleria australiensis* De Jonckheere (strain PP 397)

Characteristic	Value
CERE size (bp)	13,621
GC Content (%)	42.9
Contigs	86
BLAST coverage to *N. gruberi* rDNA (%)	46%
Average read length (bp)^*a*^	3,278.8
Total reads^*a*^	51,009
Read depth	4,876.7
N50	248
GenBank accession	OQ627480
SRA accession (Pac Bio)	SRR23735956
SRA accession (Plasmidsaurus)	SRR23736613
BioProject	PRJNA932317
BioSample	SAMN31184146
PCR primer forward (5’- >3’; nt 7,609–31)	CTC GAC TTT GAA ATT TTG TGA CG
PCR primer reverse (5’- >3’; nt 9,542–65)	CCT AGC TCA GTA CAG CCT TAA TAG
PCR conditions	95°C for 3 min then (95°C for 30 s; 58°C for 30 s; 72°C for 2 min) × 35 cycles; 72 °C × 3 min; 10°C hold

^*a*^ Values indicated represent figures after BLAST alignment against mitochondrial and *N. gruberi* rDNA as discussed in the text. When sequences were further filtered to identify sequences between 9 and 15 kbp, the average read length was 11,276.8 bp and total reads were 5,894.

**Fig 1:**
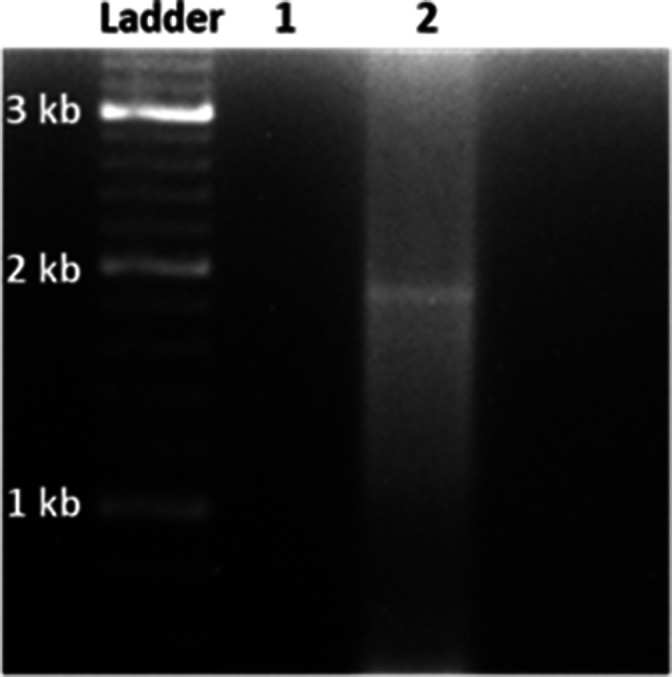
Resolution of repeat region in the *N. australiensis* sequence. To resolve the difference in repeat number between the PacBio and Oxford Nanopore consensus sequences, PCR was performed on the 7,041 bp CERE fragment obtained by digestion of the CERE with *PacI* and *XcmI*. Primers used are listed in Table 1.

To verify the predicted consensus, uncut CERE DNA was reisolated and sequenced using Oxford Nanopore technology (plasmidsaurus.com), using the V10 Chemistry Library Prep Kit, with the R9.4.1 flow cell. Guppy (v.6.3.8; SUP) was used to call bases. A 360 bp addition in a repeat region was identified in the Plasmidsaurus sequence at position 8,036, compared to the PacBio consensus. To resolve this discrepancy, PCR was performed using primers (Table 1) spanning the region. Fig. 1 indicates that three repeats are in the majority of CERE.

## Data Availability

The consensus sequence has been deposited in GenBank under accession number OQ627480. The first nucleotide of the 18S rDNA subunit has been designated as position "1." PacBio raw reads have been deposited in the NCBI Sequence Read Archive (SRA) under accession number SRR23735956. Plasmidsaurus raw reads are deposited here: SRR23736613. The BioProject and BioSample are available as follows, respectively: PRJNA932317 and SAMN31184146.
